# HPV and RNA Binding Proteins: What We Know and What Remains to Be Discovered

**DOI:** 10.3390/v16050783

**Published:** 2024-05-15

**Authors:** Sheila V. Graham

**Affiliations:** MRC-University of Glasgow Centre for Virus Research, School of Infection and Immunity, College of Medical Veterinary and Life Sciences, University of Glasgow, Glasgow G61 1QH, UK; sheila.graham@glasgow.ac.uk; Tel.: +44-141-330-6256

**Keywords:** Papillomaviruses, RNA binding proteins, RNA processing, epithelial differentiation, life cycle

## Abstract

Papillomavirus gene regulation is largely post-transcriptional due to overlapping open reading frames and the use of alternative polyadenylation and alternative splicing to produce the full suite of viral mRNAs. These processes are controlled by a wide range of cellular RNA binding proteins (RPBs), including constitutive splicing factors and cleavage and polyadenylation machinery, but also factors that regulate these processes, for example, SR and hnRNP proteins. Like cellular RNAs, papillomavirus RNAs have been shown to bind many such proteins. The life cycle of papillomaviruses is intimately linked to differentiation of the epithelial tissues the virus infects. For example, viral late mRNAs and proteins are expressed only in the most differentiated epithelial layers to avoid recognition by the host immune response. Papillomavirus genome replication is linked to the DNA damage response and viral chromatin conformation, processes which also link to RNA processing. Challenges with respect to elucidating how RBPs regulate the viral life cycle include consideration of the orchestrated spatial aspect of viral gene expression in an infected epithelium and the epigenetic nature of the viral episomal genome. This review discusses RBPs that control viral gene expression, and how the connectivity of various nuclear processes might contribute to viral mRNA production.

## 1. Introduction

Papillomaviruses (PVs) infect mainly epithelial cells to cause benign disease, i.e., warts or papillomas [1]. PVs infect a wide range of animal species and can be divided into those that infect cutaneous and those that infect mucosal epithelia. Under certain conditions, PVs can cause lesions that progress to cancer [2]. This was first investigated in cotton tail rabbit papillomavirus (CRPV), now called *Sylvilagus floridanus* papillomavirus 1 (SfPV1), infections. SfPV1 benign lesions can progress to cancer in wild rabbits, but lesions more frequently become malignant in domestic rabbits. Bovine papillomaviruses (BPVs) infect fibroblasts as well as epithelial cells, causing cutaneous papillomas but also alimentary fibropapillomas and urinary bladder tumours in cattle exposed to bracken fern in their diet and to sarcoids, a non-invasive skin tumour of horses. Over 227 human papillomaviruses (HPVs) have been identified [3]. The majority are cutaneous epithelium-infective and cause common warts or verrucas, but a subset can infect mucosal epithelia in the anogenital region and oropharynx and can cause lesions that may progress to cancer [4]. Around 5% of all human cancers are caused by infection with oncogenic HPVs. Thirteen HPVs are defined as oncogenic by the International Agency for Research on Cancer (IARC). The most prevalent HPV worldwide is HPV genotype 16 (HPV16), which causes around 55% of cancer cases. Prophylactic vaccines have been developed against this and other oncogenic HPVs [5]. These vaccines are highly efficacious, but as they are prophylactic, there is an older global population, particularly in the developing world, at risk of disease. Understanding the molecular biology of these viruses is key to developing novel antivirals and new approaches to vaccine design.

## 2. Papillomavirus Life Cycle

**Figure 1:**
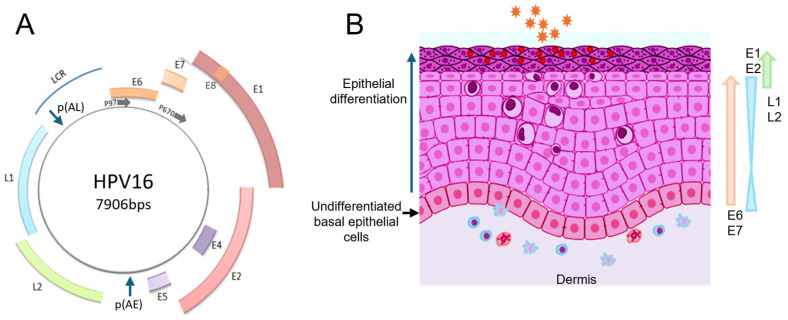
The HPV16 genome and viral life cycle. (**A**) Diagram of the HPV16 genome. Coloured arcs represent the viral open reading frames. LCR, long control region. Promoters are indicated with the letter P followed by a number and a horizontal arrow. p(AE), early polyadenylation site. p(AL), late polyadenylation site. (**B**) Diagram of an infected epithelium. Undifferentiated cells are coloured red/pink, differentiated cells are coloured pink/purple, and terminally differentiated cells at the top of the epithelium are coloured purple. Cells showing purple enlarged nuclei surrounded with halos represent koilocytes, a cytopathic effect of HPV infection. Virions (small orange stars) are produced in, and released from, the outermost layer of the epithelium. Arrows and triangles to the right-hand side indicate expression profiles of viral oncoproteins E6 and E7, replication factors E1 and E2, and capsid proteins L1 and L2. Cartoons of immune cells coloured red and blue are shown in the dermis. Partially created with Biorender.

Papillomaviruses (PVs) have double-stranded DNA genomes of around 8 kb (Figure 1A). They replicate in the nucleus, where they use the cellular DNA replication machinery to synthesise progeny viral genomes. The nuclear environment also provides the RNA processing machinery and associated regulatory factors required to synthesise viral mRNAs. PV life cycles require a differentiating epithelium for replication (Figure 1B) [6,7]. Infection occurs specifically in the dividing cells of the basal layer of the epithelium. Upon cell entry, the virus travels to the nuclear periphery and transfers its circular (episomal) genome into the host cell nucleus. The viral genome is replicated and maintained in the basal cell nuclei at around 50–100 genome copies, which are tethered to host chromosomes via the PV E2 protein and cellular BRD4 [4]. Division of an infected basal cell can lead to a daughter cell, which begins the differentiation process by moving into upper epithelial layers where vegetative viral genome replication is initiated. Replicated genomes become packaged in the nucleus once the two viral capsid proteins, L1 and L2, are synthesised. Fully formed virions are released during the disintegration of terminally differentiated keratinocytes at the epithelial surface [6,8].

During the virus life cycle, a carefully orchestrated programme of viral protein expression is tightly linked to keratinocyte differentiation (Figure 1B) [6,7]. The life cycles of the cancer-causing, or “high risk”, human alpha papillomaviruses (HR-HPVs) are best understood, and the following discussion relates to the most prevalent HR-HPV, HPV16. Upon infection and insertion into the nucleus of a basal, undifferentiated epithelial cell, the viral genome expresses its replication factors E1, a DNA helicase which engages the host cell DNA replication machinery, and E2, which binds the viral origin of replication and interacts with E1 [9]. E2 also acts as the viral transcription factor. Its main role in the early stages of the virus life cycle is to repress activity of the viral early promoter to maintain low level expression of the viral E6 and E7 oncoproteins. E6 and E7 enhance cell cycle progression [10,11] to allow HPVs to replicate in differentiated keratinocytes, which do not normally undergo cell division. Unscheduled cell division should induce apoptosis, but low levels of E6 inhibit this event by degrading the apoptosis activator, p53. These are the main early proteins of the viral life cycle. Viral proteins E4 and E5 are expressed in the early and late phases of the life cycle. Amongst other possible roles, E4 causes reassortment of the filamentous structure of differentiated keratinocytes, presumably to allow release of virus particles, while E5 regulates the host cell antiviral response [12,13]. The viral late proteins are L1, the major virus capsid protein, and L2, the minor capsid protein [14].

## 3. HPV and Cancer Progression

Persistent infection of basal epithelial cells, in concert with predisposing factors such as smoking, oral contraceptive use, and parity, can lead to cancer progression [2]. E6 and E7 are known as oncoproteins because high levels of expression induce tumour progression in cell culture, and animal, models, while raised E6 and E7 expression is the hallmark of HPV-induced tumours. This increase in oncoprotein expression may be due to increased transcription or post-transcriptional events. However, in human cells, integration of the viral episomal genome into the host genome often results in loss of the E2 coding region, meaning that E2 is no longer expressed to repress E6 and E7 expression. In addition, the location of insertion of the episomal genome into the host genome can lead to expression from a highly active cellular promoter or entry into a region of the genome with open chromatin conformation, leading to upregulated expression of the oncoproteins [15]. Finally, the oncogenes may end up under control of a heterologous 3′ UTR, resulting in mRNAs with increased stability [16]. Not all viral genomes are integrated during HPV-associate cancer progression; some remain episomal. How E6 and E7 expression is increased in this situation is less explored, but must involve changes in viral chromatin.

## 4. RNA Processing

### 4.1. Splicing

Upon transcription by RNA polymerase II (RNAPII), a nascent messenger RNA (mRNA) (pre-mRNA) emerges from the exit channel of the enzyme. The carboxy terminal domain (CTD) of RNAPII acts as a landing pad for factors that control the processes of capping, splicing, and polyadenylation of the pre-mRNA. Splicing is the process whereby introns in a pre-mRNA are removed, and exons are spliced together to form the mature mRNA. Splicing is catalysed by the spliceosome, a massive RNA/multi-protein complex which recognises splice sites and other signal sequences in the nascent RNA. The spliceosome comprises five small nuclear ribonucleoproteins particles or snRNPs: U1, U2, U4, U5, and U6 snRNP. These components of the spliceosome are reorganised at different stages of the splicing process, resulting in a sequential cleavage of the RNA at the 5′splice site, the formation of a lariat structure in the downstream intron, cleavage at the 3′ splice site, joining of the 5′ and 3′ exons, and degradation of the intron lariat.

The spliceosome recognises 5′ splice sites through U1 snRNP binding, while U2 snRNP is brought in and tethered at the so-called intron branch point through interaction with splicing factor U2AF. Then, snRNPs U4, U5, and U6 locate and interact with U1, resulting in the release of U1 and U4 snRNPs and allowing U6 snRNP to bind the 5′ splice site and interact with U2 downstream to form the intron lariat. U5 positions the 5′ and 3′ exons in the splicing reaction. Recognition of splice sites is controlled by positive and negative splicing factors. Exons contain cis-acting sequences, called exonic sequence enhancers (ESEs), while introns contain intronic sequence enhancers (ISEs), which influence splicing efficiency by binding splicing enhancing proteins. The best-studied activators of splicing are the serine-arginine rich (SR) proteins, which possess at least one RNA recognition motif (RRM) and a serine-arginine-rich domain (RS domain) which mediates interaction with other proteins [17,18,19]. Conversely, exonic sequence silencers (ESSs) or intronic sequence silencers (ISSs) bind splicing repressor proteins, the largest group of which are the hnRNP proteins. These RBPs also have RRMs and protein-binding motifs and can act in antagonism to SR proteins [20]. Splicing enhancers bind proteins that activate the use of splice sites by attracting in the splicing machinery, by stabilising components of the spliceosome, and by preventing access of splicing silencers to silencing elements through steric hinderance. Splicing silencers act by competing with splicing enhancing factors, by blocking their access, or by forming RNA loops that block splicing of certain exons. SR proteins define exons by forming a bridge between spliceosome components at 3′ and 5′ splice sites across an exon, while hnRNP proteins block these interactions.

### 4.2. Alternative Splicing

Alternative splicing is the process in cells which allows expression of more than one mRNA from a single gene by mutually exclusive exon selection, or skipping or inclusion, of exons/introns in a pre-mRNA [21]. In addition, choice of alternative splice sites can alter exon size. Although consensus sequences have been determined, 5′- and 3′-splice sites are known to be degenerate, and sometimes poorly recognised by the splicing machinery. Ambiguity in splice site recognition can lead to multiple choices of splice sites within a complex pre-mRNA [22,23]. However, the presence of ESEs/ISEs and the binding of SR proteins can enhance recognition of splice sites by the splicing machinery, while ESS/ISS and hnRNP binding can repress splice site recognition leading to exon skipping. Interestingly, the “kinetic coupling of transcription and splicing” model suggests that SR/hnRNP protein recognition of cognate sequences is regulated by RNAPII transcription rate and opportunities for recruitment to the nascent RNA [24]. Alternative splicing is also controlled by the order in which exons emerge from RNA polymerase II during transcription, the pattern of RNA binding proteins (RBPs) on the pre-mRNA, and cell signalling in response to the cellular environment [25].

### 4.3. Polyadenylation

Cleavage and polyadenylation, or mRNA 3′ end formation, is the final event during transcription of a gene. The polyadenylation machinery consists of two complexes, CPSF (composed of Fip1, CPSF-30, CPSF-73, CPSF-100, and CPSF-160), which binds an AAUAAA signal sequence in the 3′ untranslated region (3′UTR) of the pre-mRNA around 10–30 nucleotides upstream of the eventual site of mRNA cleavage, and CstF (composed of CstF-50, CstF-64, and CstF-77), which binds a GU-rich sequence located just downstream of the cleavage site. Symplekin protein acts to bring CPSF and CstF complexes together to form an RNA loop, where cleavage will occur. Polyadenylation complexes are also associated with the CTD of RNAPII through CstF-70, and are therefore connected to upstream events such as splicing. For some genes, mRNA 3′ end formation is controlled by upstream or downstream sequence elements, their RBPs, post-translation modifications of these proteins, and the protein complex formed in and around these elements [26]. Such protein–protein–RNA interaction can also regulate mRNA stability [26].

## 5. Nuclear Connectivity

**Figure 2:**
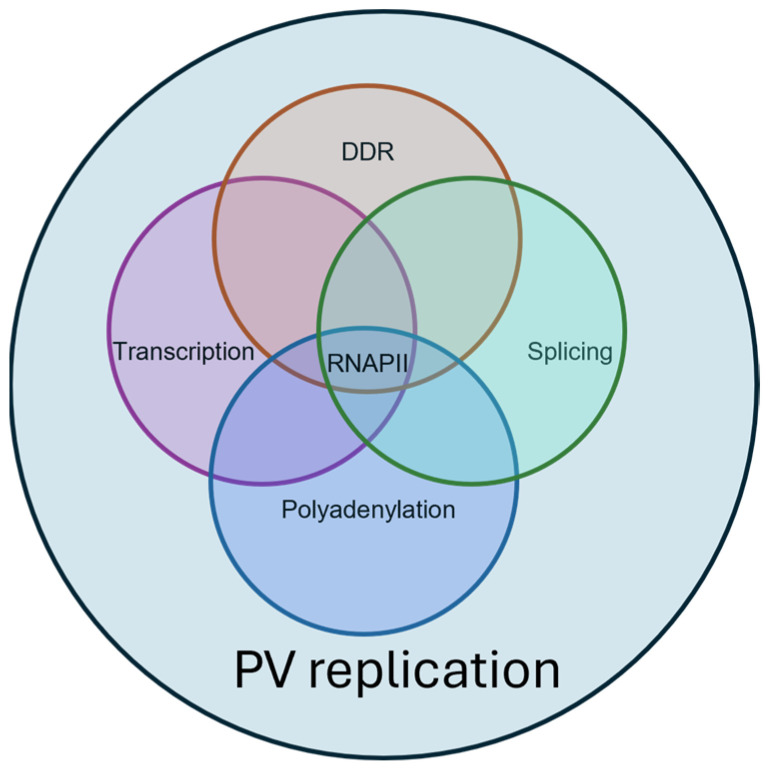
Diagram of the interrelated nuclear processes required for papillomavirus (PV) replication. Lilac circle: transcription. Blue circle: polyadenylation. Green circle: splicing. Orange circle: DNA damage response (DDR). RNAPII, RNA polymerase II.

There is now clear evidence that many nuclear processes are highly connected (Figure 2). For example, transcription initiation and elongation by RNAPII regulates splicing and polyadenylation [24], and the DNA damage response links to transcription, alternative splicing, and polyadenylation [27,28,29]. These links will be explored in more detail as regards HPV gene expression later in this article. The kinetic coupling model has emerged to explain the link between transcription and alternative splicing. Generally, an elongating RNAPII will pause sequentially at splice sites along the pre-mRNA, as it is generated, to allow spliceosome assembly. This means that each intron should be spliced out in order. However, alternative exon skipping or inclusion suggests that such ordered splicing does not always occur. Instead, the rate of elongation of RNAPII determines alternative splicing outcomes: a slow progressive RNAPII can lead to inclusion of alternative exons that display weak 3′ splice sites, since splicing factors and their regulators have more time to find the splice sites and form complexes on the nascent RNA. In contrast, fast elongation can result in exon skipping due to poor recognition of a weak splice site. Alternatively, RNA secondary structure formation over alternative exons/introns following transcription can inhibit access to splicing regulatory proteins [24]. Instead of co-transcriptional splicing, around 30% of introns are spliced out following transcription, and alternatively spliced introns may be the last to be spliced, perhaps to allow optimal recognition by RBPs [30]. There is also good evidence that changes in the recruitment of splicing factors in response to changes in the phosphorylation of the CTD of RNAPII during transcriptional elongation can regulate alternative splicing [31]. Chromatin conformation, nucleosome placement, and histone modifications can also regulate alternative splicing through changing the RNAPII elongation rate, but also by differential recruitment of splicing factor [32].

Next, different nuclear RNA processing steps are tightly linked through RNAPII and co-transcriptional RNA processing and by associated RBPs, which can be shared by several different steps in mRNA biogenesis [33,34]. For example, splicing proteins can regulate nascent RNA cleavage and polyadenylation both positively and negatively, and cleavage and polyadenylation can control splicing of upstream exons [35]. SR proteins are a good example of RBPs that participate in more than one nuclear event, because they can regulate splicing, polyadenylation, nuclear export, mRNA stability and translation, as well as genome-related functions such as RNAPII elongation, chromatin remodeling, and genome stability [16,36].

The DNA damage response (DDR) can inhibit the progression of RNAPII along the genome to avoid a molecular conflict between transcription and damage repair. Once a DNA break is detected, RNA processing is altered, and DDR-related factors are recruited to the repair site [37]. DDR reprogramming of gene expression is reliant on alternative splicing controlled by splicing regulatory factors. Some of these splicing regulatory factors have been shown to have non-splicing roles in the DDR, while other studies have demonstrated that DDR-related proteins are actually splicing factors. In summary, there is a very strong connection between DDR and alternative splicing.

## 6. RNA Binding Proteins

RNA binding proteins (RBPs) are key regulators of gene expression at every level in the RNA biogenesis and protein translation pathways. Over 1500 RBPs exist. They regulate transcription elongation, splicing, polyadenylation, nuclear export, RNA localisation, and RNA stability and translation, as well as regulating microRNA processing. In virus-infected host cells, cellular RBPs bind viral mRNAs to regulate viral gene expression. Thus, RBPs are essential components in virus replication and key contributors to viral pathogenesis.

RBPs are important players in cellular homeostasis. Therefore, it is unsurprising that many are known to be dysregulated during cancer progression, either by altered expression levels or changed subcellular location. These changes can impact the gene expression of factors involved in key cellular processes involved in tumourigenesis, such as apoptosis, angiogenesis, invasion, and metastasis.

### 6.1. RBPs and Papillomavirus Life Cycles

All papillomaviruses studied to date express multiple viral mRNAs generated by alternative splicing and differential polyadenylation. Analysis is complicated by the presence of overlapping open reading frames (ORFs), meaning that some ORFs contain introns, and the fact that most PV splice signals are suboptimal [38]. Viral RNA processing is regulated according to whether viral gene expression is in the early or late phase, which is controlled by the differentiation stage of the infected epithelium (Figure 1B). Therefore, the cellular environment and differentiation status are key regulators of the viral life cycle. There is evidence that expression of RBPs changes during epithelial differentiation and during papillomavirus-associated cancer progression [39,40,41].

### 6.2. RBPs and Splicing Regulation

The involvement of RBPs in PV life cycles and tumourigenesis was first studied for BPV RNA splicing. Early studies using in vitro splicing assays, UV crosslinking, and RNA pull-down experiments showed that two ESEs upstream of the early 3′UTR bound SRSFs 1, 2, 4, and 6, and an ESS bound PTB, U2AF, and unidentified SR proteins [42,43]. Another element bound SRSF3 in a keratinocyte differentiation-dependent manner [44]. Binding of these proteins controlled the switch between viral early and late gene expression, because the elements they bound regulated inclusion or exclusion of the viral region containing the early polyadenylation site. Enhancement of a proximal splice donor site gave rise to late mRNAs that read through the early polyadenylation site to express the L2 minor capsid protein, while enhanced selection of the distal splice donor site produced mRNAs which skipped the early polyadenylation region and the L2 ORF, leading to mRNAs encoding the major capsid protein, L1.

Several approaches have been taken to identify proteins involved in regulating HPV splicing/alternative splicing. Most studies have used transfection of plasmids expressing parts of wild-type or mutated HPV genomes in HeLa cells as a model for cervical cancer or basal epithelial, cells. RNA pull-down, UV crosslinking, and immunoprecipitation studies have been used to demonstrate RBP interactions using overexpression or siRNA depletion of specific RNA binding proteins. In recent years, a Cre-Lox system to generate recombinant episomal HPV genomes in transfected cervical cell lines has also been used.

**Figure 3:**
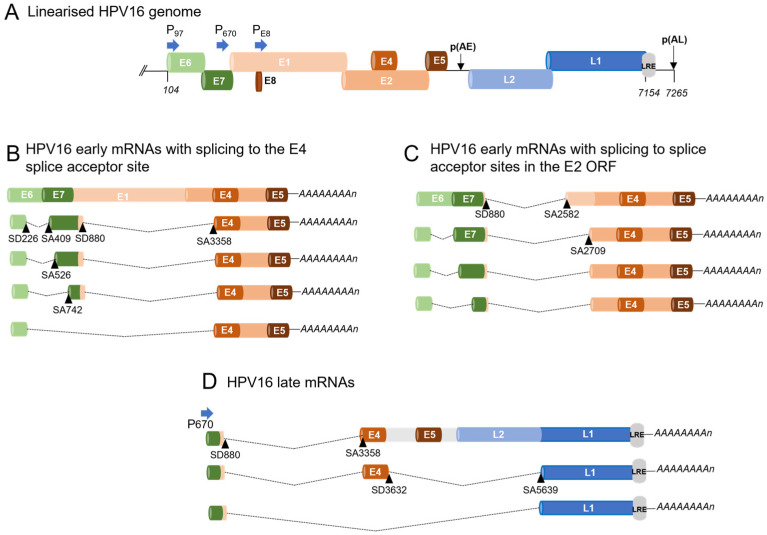
Diagram showing the splice sites used by selected HPV16 mRNAs. (**A**) Diagram of a linearised HPV16 genome showing coloured open reading frames. Promoters are indicated with blue horizontal arrows and designated “P” followed by a number. LRE, late regulatory element. p(AE), early polyadenylation site. p(AL), late polyadenylation site. (**B**) Diagrams of selected early mRNAs showing the readthrough mRNA (top diagram) and four mRNAs utilising the splice acceptor at the 5′ end of the E4 open reading frame. (**C**) Diagrams of early mRNAs utilising one of two splice acceptors located in the E2 open reading frame. Although only shown for splice acceptor 2709, it is assumed that mRNAs using each E2 splice acceptor can include any of the E6E7 region splice isoforms. (**D**) Diagrams of the main HPV16 late mRNAs. SD, splice donor site. SA, splice acceptor site. A(n), poly(A) tail. Exons are indicated with horizontal cylinders. Introns are indicated with dotted black lines. Splice donor and acceptor sites are indicated with black triangles.

**Figure 4:**
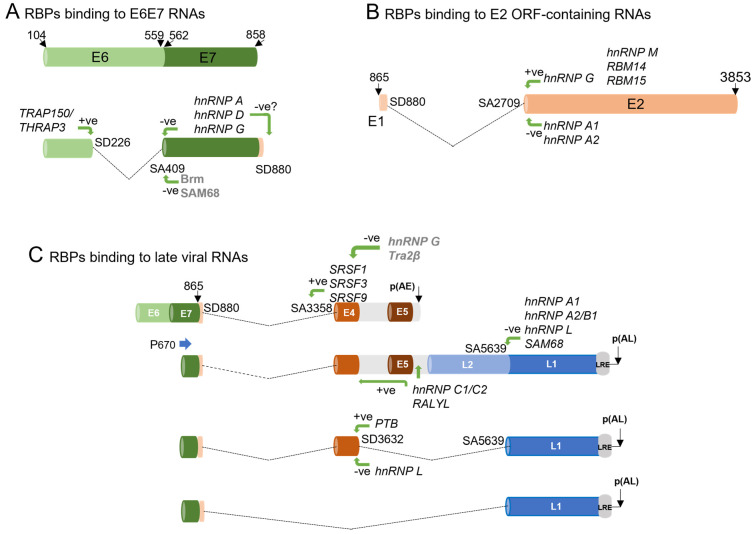
Diagrams of regions of the HPV16 RNAs shown to interact with RBPs. (**A**) RBPs interacting with exonic sequence motifs in the E6 and E7 open reading frames. (**B**) RBPs interacting with exonic sequence motifs in the E2 open reading frame. (**C**) RBPs interacting with exonic sequence motifs and the early 3′ UTR in viral late RNAs. RBPs not formally shown to bind the RNAs are shown in grey type. The location of the RBP names indicates their approximate binding position on the RNAs. Promoters are indicated with blue horizontal arrows and designated “P” followed by a number. LRE, late regulatory element. p(AE), early polyadenylation site. p(AL), late polyadenylation site. Exons are indicated with horizontal cylinders. Introns are indicated with dotted black lines. Green arrows indicate the positive (+ve) or negative (−ve) effect of various RNA binding proteins on splicing. The approximate binding positions of each of these proteins can be obtained in the review by Kajitani and Schwartz [61].

Most studies have focused on HPV16, since it is the most prevalent HPV. The HPV16 E6E7 gene region gives rise to at least four alternatively spliced mRNAs (Figure 3). The most abundant splice isoform is E6*I, where an intron from nts 226 to 409 is spliced out. This mRNA is thought to encode E7 [45]. Exon inclusion leads to a readthrough mRNA that terminates at the early polyadenylation site from which E6 is expressed. In experiments using keratinocytes transfected with a bicistronic E6E7-expressing plasmid in conjunction with siRNA depletion, splicing regulators Brm and Sam 68 were found to promote inclusion of exon 226–409 in an EGF-dependent manner, i.e., leading to expression of E6, while hnRNP A1 and A2 promoted exon exclusion which would lead to expression of E7 (Figure 4A) [46]. However, these factors were not formally proven to bind elements in the HPV16 mRNAs. Other studies on HPV16 have shown that hnRNP G and hnRNP D bind a sequence in the E7 ORF to repress production of the spliced E6*I mRNA, resulting in production of E6E7 readthrough mRNAs [47,48]. On the other hand, TRAP150/THRAP3 binds an enhancer in the E6 ORF, leading to production of the E6*I spliced RNAs from HPV16, 18, and 31 [49]. An exonic sequence silencer has been defined via mutational analysis in the HPV18 E7 ORF that inhibits the splicing event that leads to expression of E6*I by binding hnRNP A1 [50] (Figure 3). Given that relative levels of the E6- and E7-encoding mRNAs change during cancer progression [51], it will be important to understand the relative levels and contributions of these proteins to expression of the viral oncoproteins in normal versus cancer cells.

HPV E1 and E2-encoding mRNAs are expressed at low levels. Therefore, analysis of RBPs that regulate their expression during a normal infection is problematic [52]. In most viral mRNAs, most of the HPV E1 ORF is spliced out as an intron between the 5′ splice site at the beginning of the E1 ORF and the 3′ splice site at the 5′ end of the E4 ORF (Figure 3). A recent study demonstrated that hnRNP D regulates E1 inclusion as an intron in viral early mRNA; however, a binding site for this protein was not identified in the E1 region [48]. Instead, as discussed above, hnRNP D binds in the E7 ORF and could exert regulatory pressure to ignore the splice donor site at the start of the E1 gene region (Figure 4A). E2-encoding mRNAs utilise the same 5′ splice site, with splicing to one of two 3′ splice sites in the middle of the E1 gene region (Figure 4B). A recent study has revealed a splicing enhancer located in the E2 ORF, which activates splicing to the splice acceptor site at its 5′ end to allow expression of E2. hnRNP A1, hnRNP A2, hnRNP G, hnRNP M, RBM14, and RBM15 were found to bind this element. Of these, hnRNP G had an enhancing effect on splicing, while hnRNP A1 and A2 inhibited splicing [47]. Elucidating the relative contributions of these factors, and any additional RBP interactions in these ORFs, is crucial to understanding the viral life cycle since the mRNAs produced encode E1 and E2 proteins, which regulate viral transcription and replication [4].

Splicing to the 3′ splice site at the start of the E4 ORF is a very common event in both early and late viral mRNA production (Figure 3) [38]. However, like other HPV splice sites, it is of weak consensus [53]. Some seminal studies showed that SRSFs1, 3, and 9 bind to a strong ESE within the HPV16 E4 ORF to enhance recognition of the 3′ splice site located at its 5′ end (Figure 4C) [54,55,56]. The E4 ORF does not contain an ATG start codon. Its start codon is acquired from the very start of the E1 ORF through splicing from the splice donor near the beginning of the E1 gene region to the E4 splice acceptor site. E4 is the most highly expressed viral protein, suggesting that splicing to produce E4 mRNAs is tightly regulated as it is essential for the virus life cycle.

PV capsid protein expression is restricted to differentiated keratinocytes. The major capsid protein, L1, is thought to be expressed mainly from mRNAs spliced from the 5′ splice site at the 3′ end of the E4 coding region to a splice acceptor at the 5′ end of the L1 ORF. This splicing event removes, as a large intron, the early 3′ UTR and polyadenylation site, as well as the ORF encoding the L2 minor capsid protein. If this splice event does not take place, and there is readthrough of the early polyadenylation site (see below), mRNAs expressing the minor capsid protein L2 are expressed (Figure 3D). Splicing repressors hnRNP L, hnRNP A1, hnRNP A2/B1, and SAM68 have been shown to bind to elements in the L1 coding region to inhibit capsid expression, possibly by inhibiting binding of U2AF and formation of an early splicing complex at the 3′ splice site at the 5′ end of the L1 coding region (Figure 4C) [57,58,59]. PTB (hnRNP I) also controls capsid mRNA expression by negatively regulating splice inhibitory signals around the splice donor site at the end of the E4 ORF. This regulates production of the viral capsid mRNAs together with splicing silencers in the L1 ORF (Figure 4C) [60]. When cellular levels are high, PTB may alleviate repression (by other RPBs, see below) of the splice donor site at the end of the E4 ORF, leading to splicing to the splice acceptor at the 5′ end of the L1 ORF and synthesis of L1-encoding mRNAs. Alternatively, if splicing silencers are present in the L1 ORF (Figure 4C), readthrough mRNAs expressing L2 would be synthesised.

Splicing regulators RALYL and hnRNP C1/C2 bind mRNAs downstream of the HPV16 early polyadenylation site in the early 3′ UTR (Figure 4C) [62]. Overexpression of both proteins results in the production of mRNAs expressing the L1 capsid proteins. hnRNP C1 has been shown to enhance recognition of the 5′ splice site at the end of the E4 ORF, resulting in splicing out of the early 3′UTR and polyadenylation site. hnRNP L can bind to sequences around the splice donor site at the 3′ end of the E4 ORF within the early polyadenylation region, but also near the splice acceptor site at the 5′ end of the L1 ORF (Figure 4C) [63]. hnRNP L may antagonise hnRNP C1 activation of the E4 splice donor site while repressing the L1 splice acceptor site, resulting in readthrough mRNAs which do not splice out the E5 gene region and the early region 3′UTR [63]. In addition, hnRNP G and Tra2β can antagonise SR protein binding to the E4 ESE, leading to repression of expression of E4-encoding mRNAs [64].

### 6.3. RBPs Controlling HPV Early Polyadenylation

**Figure 5:**
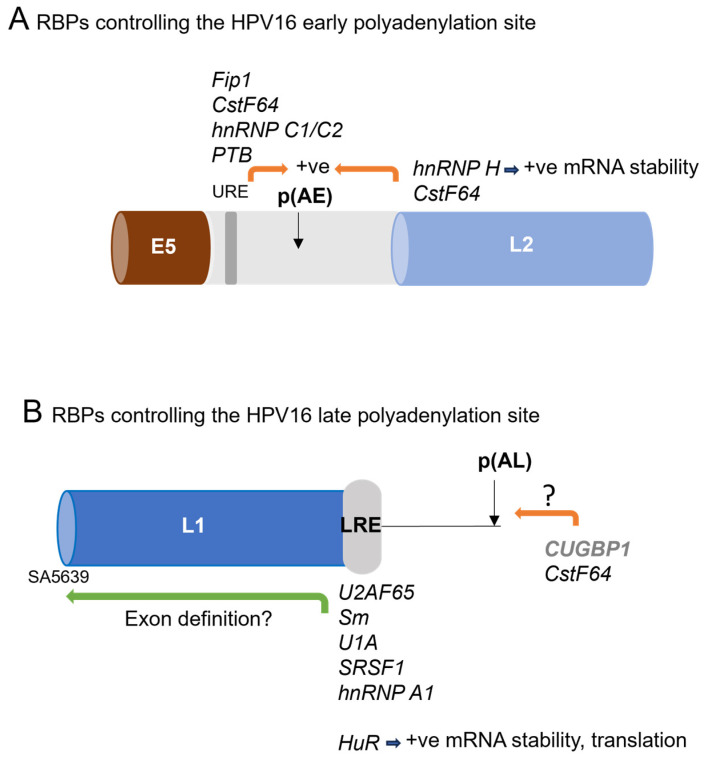
RBPs interacting with HPV16 3′ UTRs and polyadenylation sites. (**A**) RBPs that bind the HPV16 genome around the early polyadenylation site. Only the region of viral RNAs between the start of the E5 ORF and the end of the L2 ORF are shown in the diagram, which is not to scale. Open reading frames are shown by horizontal cylinders. Light grey region: early 3′UTR and polyadenylation site. Grey bar: upstream regulatory element in the early 3′ UTR. p(AE), early polyadenylation site. (**B**) RBPs that bind the late regulatory element (LRE: grey lozenge) in the HPV16 late 3′UTR. Only the L1 ORF is shown (not to scale). Green arrows, splicing regulation. Orange arrows, regulation of polyadenylation. RBPs not formally shown to bind the RNAs are shown in grey type.

HPV early gene expression terminates at the early polyadenylation site (Figure 3). HPVs possess weak consensus early pAs p(AE), requiring enhancement of recognition by the cleavage and polyadenylation machinery. The HPV16 p(AE) is the best studied to date. It has an upstream regulatory element which binds polyadenylation-enhancing RBPs such as human Fip1, cleavage stimulation factor 64 kDa subunit (CstF-64), heterogenous nuclear ribonucleoprotein (hnRNP) C1/C2, and polypyrimidine tract binding protein (PTB) Figure 5A) [65]. In addition, sequences downstream of the p(AE) in the HPV16 and HPV31 L2 ORFs (ORFs) bind splicing regulatory factor hnRNP H and CstF64 to enhance cleavage and polyadenylation (Figure 5A) [66,67,68,69].

The HPV E2 protein has three domains, a DNA binding and a transactivation domain, typical of a transcription factor, but also, similar to SR proteins, a serine-arginine-rich central hinge region [70]. E2 has been shown to have RNA binding properties [71]. It can be found in nuclear speckles, the home of SR proteins in the nucleus, and can be phosphorylated, suggesting that it may have SR protein-like properties in gene regulation [72,73,74]. E2 can bind SR proteins in HPV-infected cells, and has been shown to be part of an RNA processing interactome [75,76]. No conclusive evidence exists to show that E2 can regulate splicing, but through its transactivation domain and hinge region, E2 can control repression of the HPV early polyadenylation site by interacting with the CPSF 30 kDa subunit [77]. It remains to be seen if E2 can bind directly to viral RNAs.

### 6.4. RBPs Controlling Late Polyadenylation

**BPV1—Late Regulatory Element (LRE)**

Early experiments revealed a repressive cis-acting RNA element in the BPV1 late 3′UTR which inhibited expression of the mRNAs encoding the viral capsid proteins [78]. Having ruled out inhibition of transcription, polyadenylation, and mRNA decay to explain this phenomenon, it was suggested that the repressive element may mimic a 5′ splice site [79] through binding of a U1 snRNP complex [80]. However, the U1 70 kDa component of U1 snRNP was found to bind to poly (A) polymerase to inhibit polyadenylation [80]. It is possible that the element may have a dual function in BPV1 capsid RNA production; regulation of splicing of the L1 terminal exon, and inhibition of mRNA poly(A) addition in cells non-permissive for capsid protein expression, for example, undifferentiated epithelial cells.

**HPV1—Late Regulatory Element**

HPV1 also contains a cis-acting 57 nt element in its late 3′UTR that represses gene expression in HeLa cells [81]. The HPV1 element binds cellular proteins to repress late gene expression, potentially by restricting export to the cytoplasm [81], inducing mRNA decay [82], and inhibiting translation [83]. RBPs that interact with the element include HuR [81], a key mRNA stability regulator also known to regulate translation initiation [84], hnRNP C1/C2 [85], and poly(A) binding protein (PABP) [83], which controls polyadenylation in the nucleus and translation in the cytoplasm [86]. Experimental evidence has suggested that HuR binding to the HPV1 element was able to control translation from a CAT reporter construct because mutation of the HuR-interacting sequences reversed inhibition of translation.

**HPV16—Late Regulatory Element**

The HPV16 79 nt element overlaps the 3′ end of the L1 ORF and extends into the late 3′UTR (Figure 5B) [87,88,89]. It contains four weak 5′ splice site sequences in its 5′ portion of 47 nts and a 32 nt G/U-rich sequence in its 3′ portion. Both portions are required for repression of gene expression in HeLa cells [90]. Using mutation analysis, the weak 5′ splice sites in the 5′ portion were shown to bind Sm proteins, U1A, and U1 snRNA, but, unlike the BPV1 3′UTR element, U1 70 K binding was not detected. Importantly, these in vitro RBP binding experiments were carried out using cell extracts from undifferentiated W12 cells, which are cervical epithelial cells naturally infected with HPV16, thus taking into account the natural cellular environment of early HPV16 infection. The first RBP shown to interact with the element was U2AF 65 kDa [89,91,92], which bound the G/U-rich 3′ portion of the repressive element [89]. Binding of a U1 snRNP-like complex to the 5′ portion and U2AF to the 3′ portion was suggestive of a cross-intron splicing complex. Further work revealed the presence of SRSF1 bound to U2AF in the complex [92].

During splicing, exon–intron junction complexes at 5′ and 3′ splice sites define the exons for RNA processing [93]. Papillomavirus terminal exons are extremely long; for example, the HPV16 L1 ORF is 1.5 kb in length. The splicing complex at the 3′ splice site located at the 5′ end of the L1 ORF should interact with the cleavage and polyadenylation complex downstream of the ORF to define the exon, and the late regulatory elementwith its associated RBP complex could mimic an early splicing complex to aid cleavage and polyadenylation and enhance L1 exon definition.

Other proteins shown to bind the element in its GU-rich 3′ portion include CstF-64, although binding is relatively weak [90,91], the splicing regulator hnRNP A1 [41], and the mRNA stability regulator HuR [91,94]. The 3′ part of the element has homology to a class III HuR binding site. HuR was first shown to bind the element using HeLa cell extracts, but was later shown to bind more strongly in extracts from differentiated W12 cells compared to undifferentiated W12 cell extracts. Working on the hypothesis that HuR could stabilise late gene transcripts, the depletion of HuR in differentiated W12 cells resulted in reduced capsid protein expression, while overexpression in undifferentiated cells induced capsid protein expression [94]. These results show that HuR is a key differentiation stage-related player in HPV late gene expression by upregulating viral late RNA stability. Finally, CUG binding protein 1 (CUGBP1) was shown to repress translation of mRNAs expressed from a Renillla reporter gene containing the GU-rich 3′ portion of the HPV16 LRE in HeLa cells [95]. However, given that HPV late mRNAs have not been shown to be present in the cytoplasm of undifferentiated epithelial cells, it is not clear how this mechanism would relate to the viral life cycle.

### 6.5. RBPs Controlling HPV mRNA Stability and Translation

Capsid protein expression is tightly restricted to differentiated keratinocytes, which occupy an immune privileged site at the surface of the epithelium. They are not normally expressed in undifferentiated epithelial cells, because to do so would activate the adaptive immune response to clear the infection. The mechanism of repression of HPV16 capsid protein expression in undifferentiated epithelial cells seems to be multifactorial. As well as repression via polyadenylation and splicing, an RNA regulatory element, located at the 5′ end of the HPV16 L2 ORF, can inhibit cytoplasmic late mRNA stability [96]. This region of the L2 ORF binds hnRNP H to regulate early polyadenylation, but it is possible that hnRNP H may also control stability of the L2 readthrough RNA [67,69,97]. A second RNA regulatory element at the 3′ end of the L2 ORF has been shown to bind hnRNP K and poly(rC) binding proteins 1 and 2 to inhibit translation of L2 mRNA in vitro [98].

## 7. Epithelial Differentiation and RBPs

A key aspect of the PV life cycle is the close linkage between viral gene regulation events and epithelial differentiation. Of particular importance is the restriction of viral replication and virion assembly to the upper epithelial layers, where there is minimal immune surveillance. If the highly orchestrated patterns of viral gene expression were disrupted, it could lead to unscheduled expression of the immunogenic capsid proteins, and this would result in immune activation and clearance of the infection. Cellular differentiation-specific proteins must play an essential role in the viral life cycle by restricting capsid protein expression to the most differentiated epithelial layers. It is highly likely that cellular RBPs are expressed in a differentiation stage-specific manner, since it is known that keratinocyte differentiation changes functional groups of proteins involved in gene expression [99], and RBP levels are altered by B- and T-cell differentiation [100]. Importantly, there is evidence that HPV infection can alter RBP levels in a differentiation-specific manner. For example, SRSF1, 2, and 3 are transcriptionally upregulated by the E2 transcription factor of HPV16 and HPV31 in differentiated cervical keratinocytes [101,102,103]. This is also true of the kinase SRPK1, which controls SR protein activity by phosphorylation [104]. Therefore, it is important to establish how the levels of various RBPs known to bind and regulate PV RNA expression change during differentiation. Most studies examining RBPs interacting with or regulating HPV mRNAs have used transfections into, or cell extracts from, cervical cancer cells such as HeLa or C33a cells, or HEK293 cells, an embryonic kidney cell line, all of which have lost the capacity to differentiate. Experiments in these cells are useful as they may replicate the environment of undifferentiated epithelial cells, and can allow investigation of how RBPs regulate viral early mRNA production or repress late mRNA expression. However, since it is well recognised that many RBPs are upregulated in cancer cell lines because they control mis-regulation of cellular homeostasis [105], these cell lines probably do not express RBP levels representative of normal epithelial cells. On the other hand, results from such studies are very pertinent to HPV gene expression during tumour progression. This is particularly relevant to expression of the viral E6 and E7 oncoproteins, since their expression is directly regulated by alternative splicing, and increased levels of the oncoproteins is the direct cause of HPV-associated cancer.

## 8. Nuclear Connectivity, Papillomavirus mRNA Production and RBPs

Papillomavirus genomes are episomal, and the episomes are tightly packaged into chromatin [106]. As mentioned above, chromatin epigenetic modification and conformation and RNAPII processivity can regulate splicing and recruitment and access of RBPs to the pre-mRNA within the RNAPII “transcription factory”. The rate of RNAPII progression around this small chromatinised episomal genome is unknown. Determining RNAPII occupancy along the viral genome at early and late stages of the viral life cycle would be helpful to identify any pauses potentially linked to the various splicing events and choice of polyadenylation sites. It would also be interesting to determine if viral RNA splicing is co- or post-transcriptional, since several viral mRNAs are products of readthrough of poor consensus splice junctions, while most result from alternative splicing events. Linked to this, evidence shows that CCCTC-binding factor (CTCF)-mediated chromatin looping of HPV episomes can regulate viral alternative splicing [52]. CTCF loops can create a roadblock to RNAPII to enhance recognition of suboptimal splice sites, leading to inclusion of poorly defined exons [107]. CTCF chromatin looping changes between undifferentiated and differentiated HPV-infected epithelial cells to allow epigenetic remodelling of viral episomes and activation of viral late gene expression [108]. Linking these changes in chromatin conformation to changes in RBP interaction with HPV RNAs may reveal new insights into how viral capsid encoding RNAs are expressed.

In the past, many of the model systems used to understand HPV mRNA production and the involvement of RBPs have used plasmids expressing PV subgenomic regions under control of a heterologous promoter, often a very active promoter such as the CMV promoter. This type of approach cannot account for the natural low activity of the viral promoters, or the chromatin conformation of the viral genome. Therefore, although these experiments have revealed many RBPs that interact with HPV RNAs, how the data relate to viral mRNA production from PV episomes requires further investigation.

In infected differentiated epithelial cells, PV replication mimics DNA damage. DNA damage repair (DDR) pathways are activated, allowing recombination-mediated replication of viral genomes [109]. Therefore, DDR is key to late events in the viral life cycle. There are clear connections between DDR and alternative splicing [37]. DDR results in cellular reprogramming mediated by changes in alternative splicing. On the other hand, DDR-activated proteins are known to be splicing regulators [24,27]. In agreement with a role of DDR in late events in the PV life cycle, one study revealed that DDR inhibited use of the HPV16 early polyadenylation signal, leading to production of the late HPV16 L1 and L2 proteins [110]. Activation of DDR was found to alter HPV16 late mRNA processing from episomal genomes and induce binding of DDR factors BCLAF1 and BRCA1 and splicing factors U2AF65 and hnRNP C to viral DNA, and U2AF65 and hnRNP C to viral RNAs. Once again, the involvement of DDR in PV replication in differentiated epithelial cells underscores the need to define RBP interactions with PV RNAs during an active infection in the correct cellular environment.

## 9. Conclusions

Ultimately, a full picture of RBPs and their involvement in PV life cycles will only be obtained once CLIP-Seq data for each of the viral RNAs expressed from episomal genomes is obtained. Taking viral genome replication and the differentiation-dependent nature of the life cycle into account will be key in figuring out the various regulatory pathways to full expression of the viral transcriptome. Studies such as these could be pivotal in designing novel therapies against PV infections. For example, understanding the involvement of RBPs in restricting viral capsid protein expression to differentiated epithelial cells could lead to therapies to stimulate expression of these immunogenic proteins in the lowest epithelial layers, where they would activate patrolling immune cells (Figure 1B) to clear the infection. Alternatively, understanding RBPs essential for HPV oncogene mRNA expression in cancer cells could lead to development of a therapeutic to inhibit expression and halt the reliance of HPV-associated tumour progression on E6 and E7 expression.
